# Early Experiences of Serbian Surgeons Using No-Touch Technique for Vein Conduits in CABG Patients: A Follow-Up Study with Multi-Slice CT Angiography

**DOI:** 10.3390/medicina60091427

**Published:** 2024-08-31

**Authors:** Aleksandar Milutinović, Jelena Klajević, Igor Živković, Nemanja Milošević, Siniša Gradinac, Stefan Stanković, Želimir Antonić, Slobodan Tomić, Armin Šljivo, Miodrag Perič, Milovan Bojić, Dragana Radoičić

**Affiliations:** 1 Cardiovascular Institute “Dedinje”, 11000 Belgrade, Serbia; aleksandarm1982@yahoo.com (A.M.); jelenakljajevic@gmail.com (J.K.);; 2 Faculty of Medicine, University of Belgrade, 11001 Belgrade, Serbia; 3 Clinical Center of University of Sarajevo, 71000 Sarajevo, Bosnia and Herzegovina

**Keywords:** coronary artery disease, ischemic heart disease, early postoperative graft evaluation, no-touch vein harvesting, graft patency assessment

## Abstract

*Background and Objectives*: The saphenous vein graft (SVG) remains the most frequently used conduit worldwide, despite its common disadvantage of early graft failure. To solve the problem and reduce the SVG damage, Souza implemented a new technique where a vein is harvested with surrounding fascia and fat tissue (the so-called no-touch technique). *Materials and Methods*. A prospective study conducted from February 2019 to June 2024 included 23 patients who underwent myocardial revascularization using a no-touch vein, with follow-up control examinations using computed tomographic angiography to detect graft stenosis or occlusion. *Results*. Of the entire patient group, 17 (73.9%) were male, with a mean age of 67.39 ± 7.71 years. The mean follow-up period was 25 months. There were no major adverse cardiovascular or cerebrovascular events (MACCEs) during hospitalization, although one patient died in the hospital. Another patient died due to malignancy, but no MACCEs occurred during the follow-up period. According to multi-slice CT coronary angiography, the results were impeccable, with an astonishing 100% patency observed in all 20 IMA grafts and 58 no-touch SVGs examined. *Conclusions*. The excellent patency rate during the early follow-up period confirmed that the no-touch technique is a good option for surgical revascularization.

## 1. Introduction

Coronary artery bypass surgery (CABG) remains one of the most valuable life-saving options for myocardial revascularization [1]. Although recent trends have increased the use of multiple (MAR) or total (TAR) arterial revascularization techniques with more arterial grafts, the saphenous vein graft (SVG) continues to be the most frequently used conduit worldwide [2,3]. According to the Society of Thoracic Surgeons (STS) database, more than 89% of the patients were revascularized using the saphenous vein grafts (SVG) [4]. One of the most significant disadvantages of the SVG is early graft failure, with patency rates ranging from 75% to 86% at 5 years and 55% to 60% at 10 years [5]. The exact cause of this pathological condition remains unclear. The literature identifies two groups of etiological factors that significantly impact early or late graft occlusion. Invariable risk factors include gender, race, age, quality of coronary circulation (run-off), and vein quality. Variable factors encompass surgeon skills for proximal and distal anastomosis, surgical conduit preparation, conduit handling, and grafting site [6]. The conventional vein harvesting technique is the most commonly performed and according to its popularity and utility, it is gold standard in vein harvesting, but with serious neglected side effects. The histological and immunohistochemical examination showed that blunt surgical trauma and excessive manipulation during the harvesting process decreased endothelial integrity and function. Consequently, this accelerates thrombosis, intimal hyperplasia, and atherosclerosis in the early, intermediate, and late follow-up, respectively [7]. In an effort to address the issue of SVG damage, Souza introduced a new technique where the vein is harvested with surrounding fascia and fat tissue, known as the no-touch (NT) technique [8]. This technique begins with a longitudinal incision through the skin, where subdermal vessels are ligated to reduce the use of diathermy. Incisions are ideally made on the calf and thigh rather than at the knee level, as the vein in the knee region has a higher number of branches and is often of lower quality; plus, it is more comfortable for the patient. To expose the saphenous vein, the edges of the wound are lifted with forceps, and a plane is created around the vein using scissors, maintaining tension on the subcutaneous tissue. The vein is shielded by a thin layer of surrounding tissue and 0.5 cm of fat on either side. During vein removal, the vein is separated from its bed with scissors and diathermy, and all branches are dissected, ligated, and cut at the pedicle edge. Segments of the vein are collected from both the calf and the thigh. In the process of distal anastomosis, perivascular tissue is used to hold the vein, avoiding direct contact with surgical instruments [8]. This approach effectively preserves the microstructural integrity of the SVG and demonstrates excellent patency rates during both early and long-term follow-ups [9,10]. The NT technique for the great saphenous vein was implemented in our department three years ago by its originator.

Our study aimed to analyze initial experience with the NT harvesting technique, early one-year patency rate using multiple-slice computed coronarography (MSCT) and compare our results with those published in the literature.

## 2. Materials and Methods

**Figure 1:**
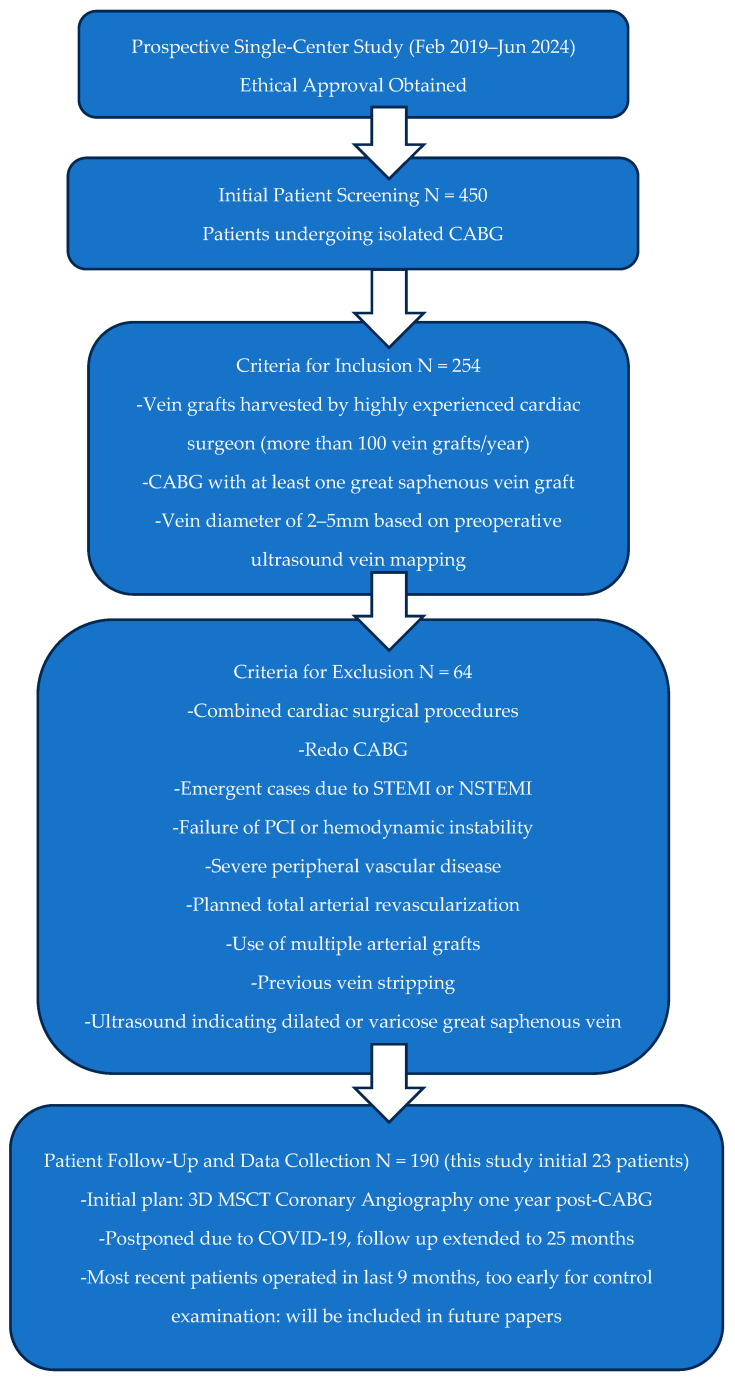
Flowchart illustrating the number of cases excluded from this study and the rationale for their exclusion.

A prospective single-center study was conducted from February 2019 to June 2024 with ethical approval from our institute. This study included patients undergoing isolated CABG who met specific criteria: patients eligible for inclusion had vein grafts harvested by a highly experienced cardiac surgeon (more than 100 vein grafts per year), underwent CABG with at least one great saphenous vein graft, and had an acceptable vein diameter of 2–5 mm based on preoperative ultrasound vein mapping. Patients meeting any of the following criteria were excluded from the study: combined cardiac surgical procedures, redo CABG, emergent cases due to STEMI or NSTEMI, failure of percutaneous coronary intervention and hemodynamic instability, severe peripheral vascular disease, planned total arterial revascularization, use of multiple arterial grafts for coronary revascularization, previous vein stripping, or ultrasound vein mapping indicating dilated or varicose great saphenous vein. A flowchart illustrating the number of cases excluded from this study and the rationale for their exclusion is presented in Figure 1.

### 2.1. Preoperative Assessment and Preoperative Ultrasound Great Saphenous Vein Mapping

The indication for the CABG procedure was made by coronary angiography examination. Significant coronary stenosis was a 50% reduction lumen or functional flow reserved test (FFR) value less than 0.75 for borderlines stenosis. Echocardiography was performed in all patients as a part of evaluation of heart function (ejection fraction and myocardial contractility), heart valve functions, and morphology of heart and great vessels.

According to our clinical protocol, vein mapping is performed one day before the CABG procedure. The vein diameter was assessed and measured at multiple sites. The acceptable diameter in our study was between 2 and 5 mm. Large tributaries, varicosities, and duplicated vein systems are detected and marked with a skin landmark.

### 2.2. CABG Procedure

Patients underwent CABG under general anesthesia. Cardiopulmonary bypass and systemic heparinization were used in all participants. Single or double clamp techniques were performed according to the surgeon’s choice. An antegrade crystalloid or blood cardioplegic solution was used for myocardial protection. Proximal and distal anastomosis of the grafts are made using standard surgical technique and distal anastomosis were sawn first in each and every patient.

### 2.3. Harvesting Technique

A complete longitudinal skin incision was made. The subcutaneous tissue dissection was performed using low power diathermy. The most important consideration is to keep the fascia and perivascular tissue intact. A vein was harvested with a 1 cm wide fat pedicle. The side branches were carefully dissected and ligated by 4-0 silk or metal clips. After reaching the needed length, the vein was removed, and in the open distal part, a small metal or plastic cannula was inserted. Forced distension or flushing using a syringe was strictly prohibited. The vein cannula was connected to the side line from the arterial cannula inserted into the ascending aorta. The heparinized blood was used for vein distension, flashing, and checking the bleeders. The pressure in the line is limited to 80–100 mmHg, and leaking side branches are identified and clipped. The subcutaneous incisions were closed by continuous Vicryl (undyed braided) suture in one or two layers depending on subcutaneous tissue thickness. The skin incisions were closed according to the surgeon’s preferences (intradermal or interrupted suture).

### 2.4. Follow-Up

All patients were contacted one year after procedure and took a part in telephone examinations. The examination was performed and major adverse cardiovascular (angina pectoris, periprocedural myocardial infarction, repeat revascularization) and cerebrovascular (transient ischemic attack, stroke) events (MACCEs) were registered. For potential leg wound complications and measuring of personal satisfaction after surgery, we used a psychometric Likert scale, the golden standard for this type of data, but that was not the topic of this paper.

The outcome of interest is occluded or stenotic (more than 50%) graft on MSCT control. Motoric disturbance was any postoperative weakness in leg strength that could be noticed during the telephone query or physical examination during MSCT control or both.

Major adverse cerebral and cardiac events (MACCEs) included the following: (1) The diagnosis of myocardial infarction was based on ECG changes (new pathological Q waves or the new left bundle branch block), new regional wall motion abnormality detected by transthoracic ultrasonography, angiographically documented graft or new native coronary artery occlusion, and elevation in cardio-specific enzymes (CK-MB five times the 99th percentile of the normal reference range and high sensitive troponin I level). (2) Angina pectoris was defined as ischemic chest pain or equivalent (e.g., arm, neck, or jaw pain or discomfort thought to be related to cardiac ischemia) without evidence of myocardial infarction. (3) Revascularization includes percutaneous coronary intervention (with or without stent implantation) and surgical revascularization. (4) Stroke was defined as a new focal neurologic deficit that persisted longer than 24 h, with acute or subacute ischemic or hemorrhagic brain lesions confirmed by computer tomography examination. Strokes were classified according to their consequences (fatal, disabling, and non-disabling).

The multiple-slice computed tomography scans were performed using a Philips Brilliance CT scanner. The scans were retrospectively ECG-gated, contrast-enhanced, and had a slice thickness of 0.67 mm. Volume rendering (VR) and multi-planar reconstruction (MPR) techniques were used for image reconstruction. Volume rendering is a type of data visualization technique that creates a three-dimensional representation of data, and mPR can be performed at oblique planes to the body or coronary arteries. The degree of the graft stenosis and occlusion were registered and analyzed by two highly experienced radiologists.

The initial plan was to perform three-dimensional MSCT coronarography a year after the CABG procedure, but the COVID-19 pandemic postponed control examination, and mean follow-up was consequently prolonged to 25 months. The majority of patients had been operated on in the last 9 months, which was too early for control; therefore, they will be part of the next paper. The limited number of patients, with only two deaths in total, was the reason for not calculating in-hospital and 30-day mortalities as separate categories. We chose to describe these two events rather than calculate them.

### 2.5. Statistical Protocol

Basic (descriptive) statistics included mean values, standard deviations, median, and interquartile range of monitored parameters. Data processing was performed using SPSS 25.0 for Windows 10.

## 3. Results

To date, 120 patients have undergone surgery using saphenous veins harvested by the NT technique. MSCT coronary angiography has been performed on the initial 23 patients from this cohort, with ongoing evaluations. In this scanned group of patients, 17 (73.9%) were male, with a mean age of 67.39 ± 7.71 years. Diabetes mellitus was documented in 7 (30.4%) patients, while preoperative myocardial infarction was detected in 13 (56.5%) patients. The other preoperative characteristics of the patients are presented in Table 1.

The cardiopulmonary bypass was used in all cases, mean bypass time being 116.55 ± 20.95 min. Cross-clamp was 75.70 ± 17.38. Apart from left internal thoracic artery (LITA), all patients received only NT SVG, while the radial artery was used as an additional graft in two patients. A vein was harvested from the thigh only in seven patients, the shank in six cases, the thigh and shank (same leg) in five patients, both shanks one patient, and both thighs in another patient. Intraoperative characteristics are presented in Table 2.

There were no MACCEs in the perioperative period. One patient died postoperatively due to respiratory insufficiency. One patient was readmitted to the hospital two days after being discharged due to mediastinal infection and died due to multiorgan insufficiency. Other postoperative complications are presented in the Table 3 and Table 4.

One patient died due to malignancy, and there were no MACCEs during the follow-up period. According to MSCT coronary angiography, all 20 IMA grafts and 58 NTSVG were patent (100%). In one of the radial arteries used for additional grafting, mild stenosis (30% in diameter) was recorded (2 cm away from the proximal anastomosis). Follow up characteristics are presented in Table 5.

## 4. Discussion

To the authors’ knowledge, this study is the first in Serbia to investigate the effectiveness of NT-SVGs in CABG patients. The results demonstrate that all IMA grafts and NT-SVGs showed 100% patency during the follow-up period, with no graft occlusions or significant stenoses observed on CT angiography. The study cohort, characterized by a high prevalence of hypertension and a substantial history of myocardial infarction, exhibited favorable outcomes with the NT technique, underscoring its potential efficacy in this high-risk population. Additionally, there were no MACCEs during the perioperative period or the follow-up. The in-hospital mortality rate was very low, with one non-cardiac death occurring during the 25-month follow-up. When comparing our study’s demographic data with similar studies [10], it is evident that our cohort exhibited a higher incidence of hypertension and had younger mean age. Additionally, it is worth noting that similar studies often do not provide detailed data regarding patient comorbidities, which may account for differences in reported outcomes [5,9].

Coronary artery revascularization represents the foremost intervention for individuals diagnosed with coronary artery disease. The determination of the optimal and safest therapeutic approach is made by a multidisciplinary team, which ensures comprehensive patient care. For cases of coronary artery disease presenting with more complex lesions, such as left main disease and multivessel disease, CABG achieves more complete revascularization compared to PCI. Furthermore, comorbidities, such as heart failure and diabetes, are always correlated with adverse clinical events, and a routine invasive strategy should be recommended [11,12]. The internal thoracic artery is widely regarded as the gold standard for surgical revascularization, with the literature documenting a patency rate exceeding 90% a decade post-procedure [7]. Despite a marked increase in the utilization of arterial grafts over the past decade, SVGs remain the most frequently employed grafts. Their continued predominance is attributed to their availability, less technically demanding harvesting procedures, and greater resistance to spasm compared to arterial grafts, rendering SVGs indispensable in surgical revascularization [13]. The primary disadvantage of conventional vein grafts is their high failure rate. In-hospital SVG failure ranges from 3% to 12%, increasing to 25% within the first year post-procedure, and only 50% to 60% remain patent at ten years [13]. Three pathophysiological mechanisms lead to SVG stenosis and occlusion: thrombosis and technical failure within the first month post-CABG, intimal hyperplasia from one month to one year, and atherosclerosis beyond one year [14]. Multiple factors contribute to the development and progression of SVG stenosis, with surgical manipulation during the harvesting process being particularly significant. Blunt trauma and excessive handling cause substantial endothelial damage, which heightens the risk of early and intermediate graft failure. Additionally, high distension pressures, reaching up to 300–600 mmHg during graft preparation, reduce ATP concentration, likely due to the disruption of the endothelial layer and vein media. This pathological condition activates regulatory molecules, promoting cell proliferation and migration into the endothelial layer [7]. Domingos Souza pioneered the technique of harvesting SVGs with the surrounding fascia and perivascular tissue intact. This innovative approach minimizes vein manipulation, preventing graft spasm and eliminating the need for distension [8]. The NT technique, by avoiding manual dilatation, enhances graft preservation compared to conventional methods [9]. Hwang et al. found less endothelial damage with NT harvesting compared to conventional techniques [15], and Tsui et al. reported lower nitric oxide release in conventionally harvested veins than in NT-harvested ones [16]. Souza et al. and others compared three saphenous vein harvesting procedures—conventional, intermediate, and NT—and found that the NT technique preserved endothelial integrity and improved CABG results [17,18]. Two randomized controlled trials also confirmed that the NT technique better preserves endothelial integrity compared to the conventional method [19,20]. Preservation of the adventitia during NT harvesting protects the vein from shear stress post-implantation into the arterial system. Beyond its mechanical role, the adventitia harbors a significant proportion of autonomic nerves and the vasa vasorum, a microvascular network crucial for gas exchange and nutrient supply to the vessel wall. Damage to the adventitia can precipitate neointimal hyperplasia and atherosclerosis, both of which are linked to vein graft failure [21]. The perivascular tissue and intact adventitia act as a “natural external stent”, providing support to the vein and mitigating the adverse effects of pulsatile stress. This protection reduces intimal hyperplasia and atherosclerosis, potentially enhancing patency rates [2]. Minimally invasive vein harvesting methods substantially preserve graft microstructural integrity, leading to improved patency rates after CABG. A prospective randomized trial demonstrated a significantly higher patency rate at 8.5 years for saphenous veins harvested using the NT technique compared to the conventional technique (90% vs. 76%) [18]. The NT technique for harvesting the saphenous vein yields significantly higher patency rates at a mean follow-up of 16 years, achieving 83% and showing results comparable to those of the LITA [10]. In our study, all 58 (100%) NTSVGs remained patent and free of stenotic lesions after a mean follow-up of 25 months. The NT technique is easily adopted, reproducible, and does not require additional equipment or resources. We advocate for the adoption and promotion of this technique, as it has the potential to enhance vein patency rates to levels comparable with arterial grafts, reduce graft attrition, and further improve the already excellent outcomes of surgical revascularization. One patient underwent sequential anastomosis performed proximally to the aorta. This type of graft is well suited for creating sequential anastomoses; however, it was employed only once among the first 23 patients. Subsequently, the use of sequential anastomoses increased in frequency, and detailed results will be presented in forthcoming publications. The primary concerns associated with this technique include potential wound healing issues [22]. Verma et al. reported a high incidence of local wound infections, swelling, and numbness with the NT technique during a 3-month follow-up [23]. In contrast, we observed no significant wound site complications, likely attributable to meticulous vein mapping, which allowed for us to avoid problematic areas of the leg. Despite these advancements, graft failure after CABG remains a challenge for a significant number of patients. For some, vein graft occlusion develops gradually and becomes clinically relevant over time, necessitating proper and timely intervention. In cases where vein graft occlusion leads to significant symptoms or ischemia, re-performing CABG or PCI may be required. The management of vein graft failure depends on the severity of the symptoms and the extent of ischemia in the myocardial region supplied by the affected graft [24]. However, there is also conflicting evidence in the literature beyond one year. The SUPERIOR SVG study found no significant difference in SVG patency between the NT and conventional harvesting techniques at one-year post-CABG, but more studies need to be conducted [25].

The primary limitation of this study is the absence of comparative analysis with saphenous veins harvested using other surgical techniques. Additionally, the relatively small sample size of the study cohort may represent a limitation. Despite this, the preliminary results from the initial year of experience with NTSVGs are promising and encourage further investigation. The excellent outcomes observed thus far provide a strong foundation for subsequent studies and evaluations. Additionally, we will also comment the Souza method. It requires a high level of surgical skill and experience, as handling the vein with minimal trauma and preparing it meticulously can be technically complex. This method can also be more time-consuming than traditional vein harvesting techniques, potentially extending the duration of the surgery, which might be a concern in emergency situations or high-risk cases. Additionally, not all patients have veins of sufficient quality or length for the no-touch technique. Veins with inadequate quality or excessive branching can still present challenges, limiting the method’s applicability. For off-pump CABG procedures, the method may introduce additional technical difficulties, as managing graft perfusion and anastomosis without cardiopulmonary bypass can be more complex.

## 5. Conclusions

The NT technique demonstrated excellent patency rates during the early follow-up period, suggesting its potential as an effective option for surgical revascularization. Further studies with larger patient cohorts are necessary to confirm these preliminary findings and to establish the long-term benefits of the NT technique.

## Figures and Tables

**Table 1:** Sex, age, BMI, NYHA classification (NYHA I–IV), ejection fraction, comorbidities, and previous cardiovascular risk factors among patients.

Preoperative Patients’ Characteristics		N (23)
Male		18 (78.3%)
Age		67.39 ± 7.71
BMI		27.51 ± 4.1
NYHA		
	I	3 (18%)
	II	14 (60.9%)
	III	4 (17.4%)
	IV	1 (4.3%)
Ejection fraction (%)		47.27 ± 7.98
Hypertension		23 (100%)
Hyperlipidemia		16 (69.6%)
Diabetes mellitus		17 (30.4%)
PVD		2 (8.7%)
CKD		3 (13%)
History of MI		13 (56.5%)
History of stroke		3 (7.5%)
Smoking		14 (35%)
Hereditary for CD		7 (39%)

BMI—body mass index; CD—cardiac disease; CKD—chronic kidney disease; COPD—chronic obstructive pulmonary disease; CVH—conventional vein harvesting; EVH—endoscopic vein harvesting; IQR—interquartile range; NT—no-touch harvesting; PVD—peripheral vascular disease.

**Table 2:** Intraoperative patients characteristics including bypasses number, ITA, GSV numbers, clamping techniques, and positions.

Intraoperative Patients Characteristics	N (23)
Number of bypasses	3 (IQR 3-3)
ITA graft (number)	22 (95.7%)
1. Pedicled	12 (52.17%)
2. Skeletonized	10 (47.83%)
GSV (number)	23 (100%)
LM disease	13 (31.7%)
LAD	
1. Number	22 (95.7%)
2. Diameter (mm)	2.3 ± 0.7
RCA	
1. Number	5 (21.7%)
2. Diameter (mm)	2.4 ± 0.55
PD	
1. Number	6 (26.1%)
2. Diameter (mm)	2.33 ± 0.5
PL	
1. Number	2 (8.7%)
2. Diameter (mm)	2.5 ± 0.7
Dg 1	
1. Number	5 (21.7%)
2. Diameter (mm)	2.4 ± 0.6
Dg 2	
1. Number	1 (4.3%)
2. Diameter (mm)	2
RI	
1. Number	3 (13%)
2. Diameter (mm)	1.67 ± 0.5
OM 1	
1. Number	12 (52.2%)
2. Diameter (mm)	1.9 ± 0.9
OM 2	
1. Number	0 (0%)
2. Diameter (mm)	
OM 3	
1. Number	3 (8.7%)
2. Diameter (mm)	3
Proximal anastomosis position	
1. Ao. Asc	22 (95.7%)
2. Other grafts	1 (4.3%)
Two-clamp technique	7 (30.4%)
Cross-clamp time (min)	75.7 ± 17.67
CPB time (min)	116.5 ± 20.922

CVH—conventional vein harvesting; CPB—cardiopulmonary bypass; Cx—circumflex artery; Dg—diagonal artery; EVH—endoscopic vein harvesting; GSV—great saphenous vein; IABP—intra-aortic balloon pump; ITA—internal thoracic artery; LAD—left anterior descending artery; NT—no-touch harvesting; OM—obtuse marginal artery; RI—ramus intermedius; RCA—right coronary artery.

**Table 3:** Postoperative patients’ characteristics including superficial sternal infection, mediastinitis, bleeding revisions, pleural effusions, new onset of atrial fibrillation, postoperative pacemaker implantation, stroke, ICU stay, overall in-hospital stay, and mortality.

Postoperative Patients’ Characteristics	N (23)
Superficial sternal infection	1 (4.3%)
Mediastinitis	1 (4.3%)
Bleeding revision	2 (8.7%)
Pleural effusion	1 (4.3%)
Atrial fibrillation	2 (8.7%)
Postoperative pacemaker implantation	1 (4.3%)
Perioperative myocardial infarction	1 (4.3%)
Stroke	0 (0%)
ICU stay (days)	2 (IQR 1–3)
In hospital stay (days)	7 (IQR 6–10)
In hospital mortality	1 (4.3%)

Abbreviations: ICU—intensive care unit.

**Table 4:** Legs wound complications.

In hospital Leg Wound Complications	N (23)
Leg wound infection	0 (0%)
Leg wound revision	1 (4.3%)
Leg pain	5 (IQR 4–5)
Leg oedema	5 (IQR 4–5)
Leg sensorial disturbance	5 (IQR 4–5)
Leg motoric disturbance	5 (IQR 5–5)
Leg wound cosmetic effect	5 (IQR 3–5)

**Table 5:** Follow-up characteristics.

Follow-Up Characteristics	N (23)
Mean follow-up period (months)	25
CT angiography (number of SVGs)	58
Graft occlusion	0 (0%)
Graft stenosis	0 (0%)
Follow-up mortality	1 (4.3%)
Cardiac death	0 (0%)
Non-cardiac death	1 (4.3%)

CT—computed tomography; SVG—saphenous vein graft.

## Data Availability

Data available upon request.
